# Healthcare Resource Use, Healthcare Costs, and Unmet Needs Among Patients Treated for EGFR-Mutated Advanced or Metastatic Non-small Cell Lung Cancer

**DOI:** 10.36469/001c.142771

**Published:** 2025-08-29

**Authors:** David Waterhouse, Iris Li, Laura Morrison, Bruno Emond, Marie-Hélène Lafeuille, Annalise Hilts, Jill Korsiak, Patrick Lefebvre, Pratyusha Vadagam, Dexter Waters

**Affiliations:** 1 OHC (Oncology-Hematology Care), Cincinnati, Ohio; 2 Johnson & Johnson, Horsham, Pennsylvania; 3 Analysis Group, Inc., Montréal, Québec, Canada

**Keywords:** advanced non-small cell lung cancer, epidermal growth factor receptor mutation, healthcare costs, healthcare resource utilization, osimertinib, platinum-based chemotherapy, tyrosine kinase inhibitors

## Abstract

**Background:**

Approximately 17% of patients with non-small cell lung cancer (NSCLC) have epidermal growth factor receptor-mutated (EGFRm) NSCLC, 84% of which are exon 19 deletions (Ex19del)/exon 21 substitutions (L858R). Unmet needs for patients treated with tyrosine kinase inhibitors (TKIs) for EGFRm (Ex19del/L858R) advanced NSCLC, including osimertinib, are relevant to US population health decision makers.

**Objectives:**

To describe healthcare resource utilization (HRU) and costs by line of therapy (LOT) among patients with EGFRm (Ex19del/L858R) advanced NSCLC initiating first-line (1L) treatment.

**Methods:**

IBM MarketScan® Research Databases (1/1/2010-1/31/2023) were used to select adult patients with advanced NSCLC initiating an EGFR-TKI during any LOT on/after 4/18/2018 (osimertinib approval; EGFRm Ex19del/L858R proxy). Per-patient-per-month (PPPM) all-cause HRU and costs were described in 1L, second-line (2L), and third-line (3L) overall and among subgroups receiving 1L osimertinib monotherapy or platinum-based chemotherapy (PBC) without immunotherapy, separately.

**Results:**

The study included 409 patients with EGFRm advanced NSCLC (mean age, 60.5 years; 70.2% female). In 1L, 72.9% initiated osimertinib-based therapy (2L, 45.9%; 3L, 41.2%), 21.0% initiated chemotherapy (2L, 30.0%; 3L, 36.5%), 4.6% initiated another EGFR-TKI (2L, 12.9%; 3L, 12.9%), and 1.5% initiated immunotherapy (2L, 11.2%; 3L, 9.4%). Overall, 170 patients (41.6%) progressed to 2L among whom 85 (50.0%) progressed to 3L. Mean LOT duration decreased with each successive LOT (1L, 10.2 months; 2L, 8.7 months; 3L, 8.0 months). Across LOTs, patients had a mean of >4 outpatient visits PPPM (1L, 4.79; 2L, 4.26; 3L, 4.40), and the 1L osimertinib monotherapy subgroup (n = 279) had a mean of 0.69 inpatient days PPPM during 1L (2L, 0.82; 3L, 0.74). Mean all-cause costs PPPM were 27 751in1L,28 971 in 2L, and 31 251in3L.Amongthe1Losimertinibmonotherapysubgroup,meanPPPMcostswere27 610 in 1L, 35 501in2L,and36 618 in 3L. Among the 1L PBC subgroup (n = 58), mean PPPM costs were 23 820in1L,24 788 in 2L, and $23 348 in 3L.

**Discussion:**

Among patients with EGFRm (Ex19del/L858R) advanced NSCLC initiating 1L, each successive LOT was shorter and more costly.

**Conclusions:**

Findings highlight the importance of using the most effective 1L treatments to delay disease progression and reduce HRU and costs.

## INTRODUCTION

Lung cancer (LC), one of the most common cancers in the United States (US), is expected to account for 12% of all new cancer diagnoses in 2024, corresponding to 234 580 new cases.^1,2^ It is also the leading cause of death due to cancer in the US, accounting for 20% of cancer deaths.^3^ Non-small cell LC (NSCLC) represents 87% of all LC diagnoses in the US,^3^ with approximately 53% of cases diagnosed as metastatic.^2^ Approximately 17% of patients with NSCLC have epidermal growth factor receptor-mutated (EGFRm) NSCLC,^4^ of whom 84% have what are referred to as common or classical *EGFR* mutations (ie, 46% exon 19 deletions [Ex19del] and 38% exon 21 codon p.Leu858Arg [L858R] substitutions) and 9% have less commonly occurring atypical or uncommon *EGFR* exon 20 insertions.^5^ Overall, patients with EGFRm (Ex19del/L858R) advanced NSCLC have a 5-year relative survival rate of only 19%.^6^

Prior to the advent of EGFR-tyrosine kinase inhibitors (TKIs), platinum-based chemotherapy (PBC) was the standard first-line (1L) treatment for EGFRm advanced NSCLC, with modest treatment outcomes.^7^ However, the increasing recognition of the importance of actionable mutations in clinical oncology has driven the rapid development of targeted treatments for patients with EGFRm advanced NSCLC. At the time of this study, 5 EGFR-TKIs had been approved by the US Food and Drug Administration (FDA) for the treatment of EGFRm (Ex19del/L858R) advanced NSCLC: the first-generation erlotinib and gefitinib, the second-generation afatinib and dacomitinib, and the third-generation osimertinib.^8^ Another third-generation TKI, lazertinib, was approved in combination with amivantamab-vmjw for EGFRm (Ex19del/L858R) locally advanced or metastatic NSCLC in August 2024.^9^

The availability of several EGFR-TKIs raises important questions regarding their optimal use and sequence for patients harboring these mutations.^8^ Studies have shown that, relative to first-generation EGFR-TKIs, 1L treatment with the standard-of-care third-generation osimertinib monotherapy has been associated with superior clinical outcomes, a more durable response reflected in the longer time on treatment before the need for second-line (2L) therapy, and a better tolerability profile.^7,10,11^

Healthcare resource utilization (HRU) and costs associated with treatments used for EGFRm (Ex19del/L858R) advanced NSCLC are of interest to population health decision makers in the US as these metrics may reflect therapeutic value. Prior work by Vanderpoel et al described real-world HRU and costs among patients receiving 1L therapy for EGFRm (Ex19del/L858R) advanced NSCLC in the US and noted that osimertinib was associated with a high number of inpatient days and high costs relative to patients who received chemotherapy.^12^ However, the study by Vanderpoel et al covered a period of less than 2 years after osimertinib approval and only evaluated outcomes during 1L therapy. This study aimed to conduct a comprehensive evaluation of treatment sequencing from 1L to third-line (3L) therapy for patients with EGFRm (Ex19del/L858R) advanced NSCLC and provide an updated description of HRU and costs by line of therapy (LOT) using contemporary data that reflect the current treatment landscape for EGFRm (Ex19del/L858R) advanced NSCLC in the US.

## METHODS

### Data Source

The IBM® MarketScan® Commercial and Medicare Supplemental Databases (Jan. 1, 2010–Jan. 31, 2023) and Multi-State Medicaid Database (Jan. 1, 2010–June 30, 2022) were used for this study. The Commercial and Medicare Supplemental databases contain data on medical and prescription drug claims, standard demographic variables, and monthly information on health plan enrollment covering all US census regions, with a higher concentration in the South and North Central (Midwest) regions. The Multi-State Medicaid Database contains data from approximately 7 million Medicaid enrollees from multiple states and includes information on inpatient/outpatient services and prescription drug claims as well as information on standard demographic variables, enrollment, long-term care, and other medical care. Data were de-identified and comply with the patient health information requirements of the Health Insurance Portability and Accountability Act; therefore, no Institutional Review Board exemption was needed.

### Sample Selection and Study Design

This study used a retrospective cohort design. Since there is no *International Classification of Diseases, Ninth/Tenth Revision, Clinical Modification* (ICD-9/10-CM) diagnosis code for EGFRm (Ex19del/L858R) or advanced NSCLC, the claims-based algorithm described in Vanderpoel et al^12^ was used to select patients with EGFRm (Ex19del/L858R) advanced NSCLC (**Supplementary Figure S1**). First, patients with at least 2 diagnoses for LC (ICD-9-CM codes: 162.2x-162.9x; ICD-10-CM codes: C34.x) during the period of continuous pharmacy and medical insurance eligibility were selected and those with small cell LC (based on a claim for 1L antineoplastic treatments used for small cell LC [ie, etoposide, irinotecan, or topotecan]) were excluded. In the remaining population, a washout period of at least 12 months of continuous pharmacy and medical insurance eligibility prior to the first observed LC diagnosis was imposed to confirm that it was the first LC diagnosis. Patients were considered as having advanced NSCLC if they initiated 1L therapy with a guideline-recommended treatment regimen for advanced NSCLC (**Supplementary Table S1**) or had a diagnosis for metastatic disease (ICD-9-CM codes: 196.x, 197.x, 198.x, 209.7; ICD-10-CM codes: C77.x, C78.x, C79.x, C7B.x) within 30 days following the first LC diagnosis. For patients who were selected based on a diagnosis for metastatic disease, the subsequent initiation of a 1L therapy any time before the end of continuous pharmacy and medical insurance eligibility or data availability was also required. Finally, patients included in the study were required to initiate an EGFR-TKI (ie, gefitinib, erlotinib, afatinib, osimertinib, and dacomitinib) during any LOT on or after April 18, 2018 (ie, the date of osimertinib FDA approval), which defined the index date and served as a proxy for EGFRm (Ex19del/L858R) status. Patients with at least 1 diagnosis for any other cancer prior to the first LC diagnosis were excluded. Among patients who did not have a diagnosis for metastatic disease within 30 days following the first LC diagnosis, those with at least 1 claim for LC-related surgery in the period of continuous pharmacy and medical insurance eligibility prior to the index date were excluded to avoid selecting patients who received treatment for early-stage NSCLC (ie, patients with stage I–IIIA). The baseline period spanned the 12-month period prior to the initiation of 1L therapy. Patients were observed during the observation period, which spanned until the earliest of the end of continuous insurance eligibility or the end of data availability (ie, Jan. 31, 2023, for Commercial and Medicare Supplemental Databases and June 30, 2022 for the Multi-State Medicaid Database).

### Identification of Lines of Therapy

The first claim for any guideline-recommended treatment for advanced NSCLC after the first LC diagnosis was identified as the initiation of 1L therapy. All guideline-recommended treatments for advanced NSCLC received within 21 days following the initiation of 1L therapy were included in the 1L therapy regimen. A period of 21 days was chosen as it represents 1 cycle of therapy for common regimens in LC, including the preferred guideline-recommended treatment for advanced NSCLC pembrolizumab plus carboplatin/cisplatin plus pemetrexed.^13^ In addition, since adjustment to therapy may occur following the first cycle due to toxicity or adverse events, the 21-day window precludes capturing this adjustment as being part of the current LOT. The end of 1L therapy was defined as the day prior to the initiation of a new antineoplastic treatment that was not part of the 1L regimen, at the resumption of the same monotherapy or combination therapy regimen after a >90-day gap (ie, re-treatment), or at the end of the observation period if there was no additional LOT observed. The date of initiation of the next LOT (ie, 2L and 3L) was defined as the date of initiation of a new antineoplastic treatment that was not part of the prior LOT (ie, 1L and 2L, respectively) or at the resumption of the same treatment regimen after a >90-day gap (ie, re-treatment). All LOTs following 1L therapy were generated in a similar manner to 1L.

### Study Measures and Outcomes

Patient demographics and clinical characteristics were described during the baseline period and treatment sequencing (ie, regimens utilized for each LOT) was described during the observation period. All-cause HRU (ie, inpatient, outpatient, and emergency department visits) and healthcare costs (ie, inpatient costs, outpatient [antineoplastic and non-antineoplastic] costs, emergency department and other costs, and pharmacy costs [including costs for oral EGFR-TKIs]), incurred during the observation period were described per patient per month (PPPM) by LOT. Healthcare costs were reported from a payer’s perspective and adjusted to 2022 US dollars based on the medical care component of the Consumer Price Index.^14^ Baseline characteristics, HRU, and healthcare costs were described among all patients and separately for the subgroup of patients treated with osimertinib monotherapy in 1L therapy and the subgroup of patients treated with PBC without immunotherapy in 1L therapy. All measures were reported using means and SD for continuous variables and frequencies and proportions for categorical variables. Mean differences (MDs) and corresponding 95% confidence intervals (CIs) were reported when assessing the increase in costs with each subsequent LOT for the overall cohort and when comparing costs between the 1L osimertinib and 1L PBC without immunotherapy subgroups.

## RESULTS

### Study Population

This study included 409 patients with EGFRm (Ex19del/L858R) advanced NSCLC (**Table 1**). The mean age of patients at 1L therapy initiation was 60.5 years, 70.2% were female, and the majority (68.0%) had commercial health insurance. A subgroup of 279 patients were treated with osimertinib monotherapy in 1L therapy, while a subgroup of 58 patients was treated with PBC without immunotherapy in 1L therapy (**Table 1**). The characteristics of patients in the subgroups were similar to those in the overall cohort, with the exception of brain metastases, which were present in 44.4% of patients treated with osimertinib monotherapy in 1L therapy and 25.9% of patients treated with PBC without immunotherapy in 1L therapy, and the use of LC-related treatments before the initiation of 1L therapy, including corticosteroids (osimertinib, 77.8%; PBC, 98.3%), radiotherapy (osimertinib, 36.6%; PBC, 72.4%), and surgery (osimertinib, 14.3%; PBC, 46.6%).

**Table 1.** Baseline Demographic and Clinical Characteristics, HRU, and Costs^a^

	**Overall (N = 409)**	**Treated With Osimertinib Monotherapy in 1L Therapy (N = 279)**	**Treated With PBC Without Immunotherapy in 1L Therapy (N = 58)**
Demographic characteristics			
Age (y), mean ± SD [median]	60.5 ± 10.0 [60.0]	60.8 ± 10.3 [61.0]	58.9 ± 7.1 [59.0]
Female, n (%)	287 (70.2)	196 (70.3)	41 (70.7)
Year of index date, n (%)			
2018	76 (18.6)	49 (17.6)	11 (19.0)
2019	95 (23.2)	68 (24.4)	11 (19.0)
2020	75 (18.3)	49 (17.6)	10 (17.2)
2021	92 (22.5)	65 (23.3)	14 (24.1)
2022	71 (17.4)	48 (17.2)	12 (20.7)
Insurance plan, n (%)			
Commercial	278 (68.0)	189 (67.7)	43 (74.1)
Medicare Supplemental	95 (23.2)	70 (25.1)	8 (13.8)
Medicaid	36 (8.8)	20 (7.2)	7 (12.1)
Baseline clinical characteristics			
Quan-CCI, mean ± SD [median]	7.4 ± 2.1 [7.0]	7.4 ± 2.0 [7.0]	7.0 ± 1.8 [7.0]
Time between first LC diagnosis and initiation of 1L (mo), mean ± SD [median]	1.7 ± 5.4 [1.0]	1.5 ± 4.5 [1.0]	2.6 ± 9.6 [1.2]
Time between first LC diagnosis and initiation of first EGFR-TKI (mo), mean ± SD [median]	3.8 ± 8.7 [1.1]	1.5 ± 4.5 [1.0]	14.1 ± 15.5 [7.2]
LC-related treatment received before initiation of 1L, n (%)			
Corticosteroids	334 (81.7)	217 (77.8)	57 (98.3)
Radiotherapy	167 (40.8)	102 (36.6)	42 (72.4)
Surgery^b^	77 (18.8)	40 (14.3)	27 (46.6)
LC-related treatment received within 30 days before initiation of 1L, n (%)			
Radiotherapy	133 (32.5)	98 (35.1)	20 (34.5)
Surgery^b^	39 (9.5)	26 (9.3)	7 (12.1)
Presence of brain or cerebral meninges metastases, n (%)	165 (40.3)	124 (44.4)	15 (25.9)
Baseline monthly all-cause HRU and costs, mean ± SD [median]			
No. of inpatient admissions	0.05 ± 0.06 [0.08]	0.05 ± 0.06 [0.08]	0.05 ± 0.05 [0.04]
No. of days with outpatient services	1.96 ± 2.13 [1.58]	1.93 ± 2.07 [1.58]	1.78 ± 0.90 [1.58]
No. of days with emergency room visits	0.09 ± 0.15 [0.08]	0.10 ± 0.17 [0.08]	0.06 ± 0.10 [0.00]
Total healthcare costs (2022 USD)	5146 ± 5358 [3608]	5475 ± 5763 [3692]	4315 ± 3718 [3524]
Medical costs	5012 ± 5285 [3519]	5343 ± 5689 [3616]	4238 ± 3716 [3412]
Inpatient costs	2617 ± 4581 [611]	2842 ± 5050 [632]	2183 ± 3462 [0]
Outpatient costs	2183 ± 2284 [1444]	2262 ± 2349 [1432]	1967 ± 1237 [1719]
Emergency room and other costs^c^	211 ± 512 [14]	240 ± 524 [17]	87 ± 207 [7]
Pharmacy costs	134 ± 392 [36]	131 ± 375 [33]	77 ± 123 [36]

Abbreviations: 1L, first-line; CCI, Charlson Comorbidity Index; EGFR, epidermal growth factor receptor; HRU, healthcare resource utilization; LC, lung cancer; mo, months; PBC, platinum-based chemotherapy; SD, standard deviation; TKI, tyrosine kinase inhibitor; USD, US dollars; y, years.

^a^Demographic characteristics were evaluated on the index date, clinical characteristics, except for Quan-CCI, were evaluated any time during the period of continuous insurance eligibility prior to the initiation of 1L therapy, and Quan-CCI, monthly all-cause HRU and costs were evaluated during the 12-month baseline period.

^b^Includes segmentectomy, lung lobectomy, and pneumectomy.

^c^Other costs are related to all services other than outpatient, inpatient, and emergency room services, including durable medical equipment as well as dental and vision care.

### Treatment Sequencing

**Figure 1.**
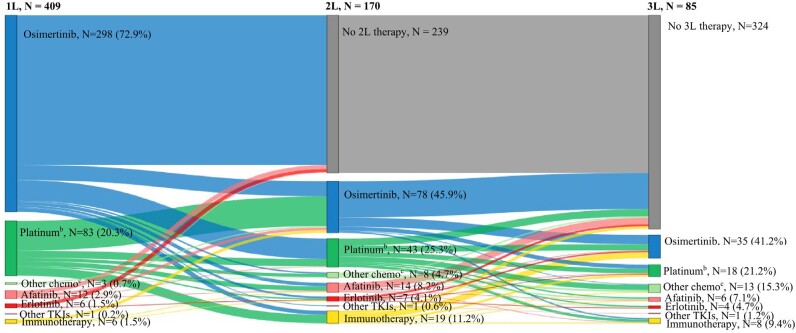
Treatment Sequencing from 1L to 3L^a^ Abbreviations: 1L, first line; 2L, second line; 3L, third line; LOT, line of therapy; PBC, platinum-based chemotherapy; TKI, tyrosine kinase inhibitor. ^a^Treatment proportions are based on the number of patients still treated (ie, excluding those who did not receive 2L or 3L therapy). ^b^PBC regimens include PBC with or without immunotherapy or other types of chemotherapy. ^c^Other chemotherapy regimens include those without PBC.

In 1L therapy, 298 of the 409 patients (72.9%) in the overall cohort initiated osimertinib-based therapy (including osimertinib monotherapy and osimertinib plus chemotherapy combination therapy; 2L, 45.9%; 3L, 41.2%), 21.0% initiated chemotherapy with or without immunotherapy (2L, 30.0%; 3L, 36.5%), 4.6% initiated a non-osimertinib EGFR-TKI (2L, 12.9%; 3L, 12.9%), and 1.5% initiated immunotherapy (2L, 11.2%; 3L, 9.4%; **Figure 1**). Subsequently, 170 of the 409 patients (41.6%) progressed to 2L therapy and of those, 85 patients (50.0% of those who received 2L therapy or 20.8% of the overall cohort) further progressed to 3L therapy; the mean duration for each successive LOT was shorter at 10.2 months in 1L, 8.7 months in 2L, and 8.0 months in 3L (**Table 2**). A total of 279 patients (68.2%) were treated with osimertinib monotherapy in 1L therapy (mean LOT duration, 11.9 months), 67 (24.0%) of them progressed to 2L therapy (mean LOT duration, 7.4 months) and 33 (49.2%) among those further progressed to 3L therapy (mean LOT duration, 7.0 months). A total of 58 patients (14.2%) were treated with PBC without immunotherapy in 1L therapy with a mean LOT duration of 5.9 months. Since all patients were required to initiate a TKI in any LOT to be included in the study, all the patients treated with PBC in 1L therapy progressed to 2L therapy (mean LOT duration, 10.0 months), of whom 33 (56.9%) further progressed to 3L therapy (mean LOT duration, 9.4 months).

**Table 2.** All-Cause HRU During Each LOT

	**Overall**			**Treated With Osimertinib Monotherapy in 1L Therapy**			**Treated With PBC Without Immunotherapy in 1L Therapy**		
**1L (N = 409)**	**2L (N = 170)**	**3L (N = 85)**	**1L (N = 279)**	**2L (N = 67)**	**3L (N = 33)**	**1L (N = 58)**	**2L (N = 58)**	**3L (N = 33)**	
Duration from initiation of 1L therapy until end of observation, including subsequent LOT (mo), mean ± SD [median]	16.4 ± 14.0 [12.1]	22.9 ± 15.3 [18.3]	26.5 ± 14.1 [25.6]	14.9 ± 12.9 [11.1]	23.5 ± 14.1 [19.3]	26.3 ± 13.0 [25.7]	23.7 ± 17.8 [16.1]	23.7 ± 17.8 [16.1]	27.6 ± 16.2 [25.7]
Duration of LOT^a^ (mo), mean ± SD [median]	10.2 ± 10.3 [6.7]	8.7 ± 9.4 [5.4]	8.0 ± 6.9 [5.5]	11.9 ± 10.7 [8.6]	7.4 ± 8.9 [4.4]	7.0 ± 4.8 [4.9]	5.9 ± 9.5 [3.4]	10.0 ± 9.7 [6.4]	9.4 ± 8.7 [7.1]
Patients with HRU, n (%)									
≥1 outpatient service	405 (99.0)	167 (98.2)	84 (98.8)	275 (98.6)	67 (100.0)	32 (97.0)	58 (100.0)	56 (96.6)	33 (100.0)
≥1 inpatient admission	114 (27.9)	57 (33.5)	28 (32.9)	82 (29.4)	28 (41.8)	11 (33.3)	15 (25.9)	12 (20.7)	9 (27.3)
≥1 emergency room visit	148 (36.2)	65 (38.2)	28 (32.9)	106 (38.0)	25 (37.3)	10 (30.3)	14 (24.1)	15 (25.9)	11 (33.3)
Monthly HRU, mean ± SD [median]									
No. of days with outpatient services^b^	4.79 ± 3.85 [3.68]	4.26 ± 2.83 [3.39]	4.40 ± 2.44 [3.99]	3.95 ± 3.12 [3.22]	5.26 ± 3.02 [4.35]	4.49 ± 2.58 [4.19]	7.87 ± 3.92 [7.60]	3.21 ± 1.85 [2.90]	4.19 ± 2.60 [3.66]
No. of inpatient admissions	0.08 ± 0.19 [0.00]	0.09 ± 0.21 [0.00]	0.13 ± 0.28 [0.00]	0.08 ± 0.20 [0.00]	0.13 ± 0.26 [0.00]	0.10 ± 0.18 [0.00]	0.05 ± 0.10 [0.00]	0.06 ± 0.19 [0.00]	0.10 ± 0.24 [0.00]
No. of days of inpatient stay among all patients	0.63 ± 2.02 [0.00]	0.62 ± 1.91 [0.00]	0.98 ± 2.73 [0.00]	0.69 ± 2.19 [0.00]	0.82 ± 1.91 [0.00]	0.74 ± 2.06 [0.00]	0.31 ± 0.69 [0.00]	0.55 ± 2.42 [0.00]	0.47 ± 1.29 [0.00]
No. of days of inpatient stay, among patients with ≥1 inpatient admission	2.27 ± 3.31 [1.12]	1.84 ± 2.95 [0.82]	2.96 ± 4.13 [1.11]	2.36 ± 3.53 [1.20]	1.97 ± 2.56 [0.98]	2.21 ± 3.17 [1.06]	1.19 ± 0.92 [0.97]	2.68 ± 4.92 [1.31]	1.72 ± 2.05 [0.93]
No. of days with emergency room visits	0.13 ± 0.30 [0.00]	0.15 ± 0.42 [0.00]	0.10 ± 0.19 [0.00]	0.12 ± 0.29 [0.00]	0.14 ± 0.32 [0.00]	0.09 ± 0.17 [0.00]	0.11 ± 0.30 [0.00]	0.06 ± 0.14 [0.00]	0.10 ± 0.18 [0.00]
Patients with supportive care, n (%)									
Claims for pain management treatments^c^	267 (65.3)	115 (67.6)	65 (76.5)	152 (54.5)	56 (83.6)	30 (90.9)	56 (96.6)	30 (51.7)	20 (60.6)
Claims for use of respiratory support care	23 (5.6)	14 (8.2)	11 (12.9)	12 (4.3)	3 (4.5)	1 (3.0)	3 (5.2)	2 (3.4)	3 (9.1)
Claims for G-CSF/GM-CSFs	20 (4.9)	8 (4.7)	5 (5.9)	2 (0.7)	8 (11.9)	7 (21.2)	13 (22.4)	3 (5.2)	3 (9.1)
Claims for ESAs	0 (0.0)	2 (1.2)	2 (2.4)	0 (0.0)	1 (1.5)	1 (3.0)	0 (0.0)	1 (1.7)	1 (3.0)

Abbreviations: 1L, first-line; 2L, second-line; 3L, third-line; ESA, erythropoiesis-stimulating agent; G-CSF/GM-CSF, granulocyte or granulocyte-macrophage colony-stimulating factor; HRU, healthcare resource utilization; LOT, line of therapy; mo, months; PBC, platinum-based chemotherapy; SD, standard deviation.

^a^Duration of the LOT was defined as the time from the date of initiation of the LOT until the day preceding the initiation of the following LOT. Patients who did not initiate a next LOT were censored at the end of their observation period.

^b^The distribution for the monthly number of days with outpatient services during 1L for patients treated with osimertinib monotherapy in 1L therapy (n=279) was, n (%), between 0 and <2 days, 66 (23.7); between 2 and <5 days, 149 (53.4); between 5 and <8 days, 42 (15.1); ≥8 days, 22 (7.9).

^c^Included amitriptyline, carbamazepine, citalopram, clonidine, clomipramine, clonazepam, dexamethasone, duloxetine, gabapentin, hydromorphone, imipramine, ketamine, morphine, nortriptyline, oxycodone, and pregabalin.

### Healthcare Resource Utilization

In the overall cohort, the mean number of days PPPM with inpatient stays during 1L therapy was 0.63 (2L, 0.62; 3L, 0.98; **Table 2**). An inpatient admission during 1L therapy was observed in 27.9% of patients (2L, 33.5%; 3L, 32.9%) and among them, the mean number of days PPPM of inpatient stays during 1L therapy was 2.27 (2L, 1.84; 3L, 2.96). Among the subgroup of patients who received osimertinib monotherapy in 1L therapy, the mean number of days PPPM of inpatient stays during 1L therapy was 0.69 (2L, 0.82; 3L, 0.74), 29.4% of patients had an inpatient admission during 1L therapy (2L, 41.8%; 3L, 33.3%) and among them, the mean number of days PPPM of inpatient stays during 1L therapy was 2.36 (2L, 1.97; 3L, 2.21). In the subgroup of patients who received PBC without immunotherapy in 1L therapy, the mean number of days PPPM of inpatient stays during 1L therapy was 0.31 (2L, 0.55; 3L, 0.47), 25.9% of patients had an inpatient admission during 1L therapy (2L, 20.7%; 3L, 27.3%) and among them, the mean number of days PPPM of inpatient stays during 1L therapy was 1.19 (2L, 2.68; 3L, 1.72).

Across all LOTs, almost all patients (97%-100%) in the overall cohort as well as the 2 subgroups had an outpatient visit during the observation period and on average, patients had multiple outpatient visits each month. Specifically, the mean number of days PPPM with outpatient services during 1L therapy was 4.79 in the overall cohort (2L, 4.26; 3L, 4.40), 3.95 among patients treated with osimertinib monotherapy in 1L therapy (2L, 5.26; 3L, 4.49), and 7.87 among patients treated with PBC without immunotherapy in 1L therapy (2L, 3.21; 3L, 4.19; **Table 2**).

In the overall cohort, the mean number of days PPPM with specific service provider types on outpatient services during 1L therapy was 1.77 for a provider type defined as an acute care hospital, 1.00 for a radiologist and imaging center, 0.69 for an oncologist, 0.44 for a general physician, and 0.39 for a laboratory. Among patients treated with osimertinib monotherapy in 1L therapy, 23.7% of patients had fewer than 2 days with outpatient services per month during 1L, 53.4% of patients had between 2 and fewer than 5 days, 15.1% of patients had between 5 and fewer than 8 days, and 7.9% of patients had at least 8 days with outpatient services per month during 1L. During 1L treatment, patients treated with osimertinib monotherapy in 1L predominantly received outpatient services with a diagnosis code for LC or metastatic cancer, while in an inpatient setting, the most commonly observed principal diagnosis was for LC or diseases of the circulatory system. The most frequent outpatient procedures during 1L among patients treated with osimertinib monotherapy in 1L consisted of evaluation and management visits and diagnostic/monitoring services, while the most frequent inpatient procedures were primarily related to inpatient or observation care for the evaluation and management of a patient. The proportion of patients with claims for pain management treatments increased with each LOT in the overall cohort (1L, 65.3%; 2L, 67.6%; 3L, 76.5%) and among patients receiving osimertinib monotherapy in 1L therapy (1L, 54.5%; 2L, 83.6%; 3L, 90.9%). Among patients who received PBC without immunotherapy in 1L therapy, the proportion of patients with claims for pain management treatments was highest during 1L therapy relative to subsequent lines (1L, 96.6%; 2L, 51.7%; 3L, 60.6%; **Table 2**).

### Healthcare Costs

**Figure 2.**
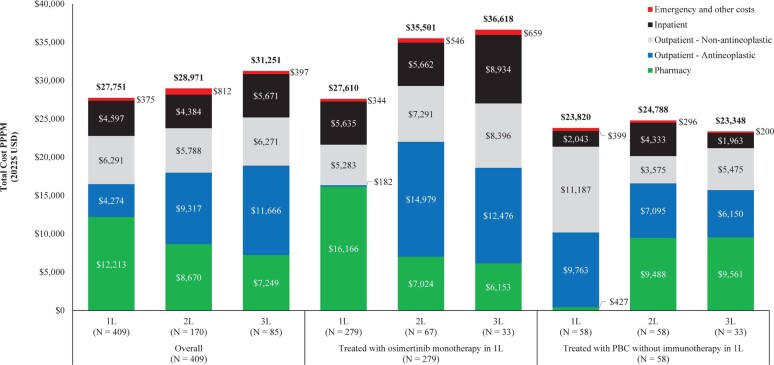
All-Cause Healthcare Costs PPPM, by LOT Abbreviations: 1L, first line; 2L, second line; 3L, third line; LOT, line of therapy; PBC, platinum-based chemotherapy; PPPM, per patient per month; USD, US dollars.

The mean all-cause total healthcare costs PPPM numerically increased with each successive LOT in the overall cohort (1L, $27 751; 2L, $28 971; 3L, $31 251; **Table 3**) and more notably among patients treated with osimertinib monotherapy in 1L therapy (1L, $27 610; 2L, $35 501; 3L, $36 618; **Table 4**). In the overall cohort, the mean increase (95% CI) in all-cause total healthcare costs PPPM was $1220 (−$3190, $5630) in 2L relative to 1L and $2280 (−$4710, $9270) in 3L relative to 2L. Mean all-cause total healthcare costs PPPM were numerically lower among the subgroup of patients who received PBC without immunotherapy in 1L therapy relative to the overall cohort and those treated with osimertinib monotherapy in 1L therapy and remained similar across LOTs (1L, $23 820; 2L, $24 788; 3L, $23 348; **Figure 2**). The MD (95% CI) in all-cause total healthcare costs PPPM between the subgroup of patients who were treated with osimertinib monotherapy in 1L therapy relative to those who received PBC without immunotherapy in 1L therapy was $3790 (−$1744, $9324) in 1L, $10 713 ($1607, $19 819) in 2L, and $13 270 ($671, $25 869) in 3L (**Table 4**). In the overall cohort, inpatient costs accounted for 16.6% ($4597 PPPM) and outpatient costs accounted for 38.1% ($10 565 PPPM) of the all-cause total healthcare costs in 1L therapy. In 2L therapy, inpatient costs accounted for 15.1% ($4384 PPPM) and outpatient costs increased to 52.1% ($15 105 PPPM) of the all-cause total healthcare costs. In 3L therapy, inpatient costs accounted for 18.1% ($5671) and outpatient costs increased to 57.4% ($17 937 PPPM) of the all-cause total healthcare costs. Pharmacy costs decreased by LOT from $12 213 in 1L to $8670 in 2L to $7249 in 3L, representing 44.0%, 29.9%, and 23.2% of the total costs in each LOT, respectively.

**Table 3.** All-Cause Costs During Each LOT

**Monthly All-Cause Total Healthcare Costs, 2022 USD, Mean ± SD [Median]**	**1L [A] (N = 409)**	**2L [B] (N = 170)**	**B vs A,^a^ MDs (95% CIs)**	**3L [C] (N = 85)**	**C vs B,^a^ MDs (95% CIs)**
Duration of LOT^b^ (mo), mean ± SD [median]	10.2 ± 10.3 [6.7]	8.7 ± 9.4 [5.4]		8.0 ± 6.9 [5.5]	
Total healthcare costs	27 751 ± 24 709 [22 340]	28 971 ± 24 632 [21 090]	1220 (−3190; 5630)	31 251 ± 27 890 [22 594]	2280 (−4710; 9270)
Medical costs	15 538 ± 25 694 [7118]	20 301 ± 25 858 [9673]	4763 (147; 9379)	24 002 ± 27 605 [14 584]	3701 (−3338; 10 740)
Outpatient costs	10 565 ± 15 437 [4384]	15 105 ± 20 127 [6677]	4540 (1165; 7915)	17 937 ± 22 663 [9895]	2832 (−2857; 8521)
Antineoplastic drug + related to administration costs	4274 ± 13 660 [0]	9317 ± 19 346 [0]	5043 (1848; 8238)	11 666 ± 24 329 [200]	2349 (−3585; 8283)
Other outpatient costs	6291 ± 9403 [3325]	5788 ± 9768 [3039]	−503 (−2231; 1225)	6271 ± 12 533 [2476]	483 (−2559; 3525)
Inpatient costs	4597 ± 20 665 [0]	4384 ± 13 666 [0]	−213 (−3082; 2656)	5671 ± 15 104 [0]	1287 (−2525; 5099)
Emergency room + other costs^c^	375 ± 1159 [0]	812 ± 4702 [0]	437 (−279; 1153)	394 ± 1714 [0]	−418 (−1213; 377)
Pharmacy costs	12 213 ± 8132 [14 776]	8670 ± 9728 [6662]	−3543 (−5204; −1882)	7249 ± 7093 [5318]	−1421 (−3522; 680)

Abbreviations: 1L, first-line; 2L, second-line; 3L, third-line; CI, confidence interval; LOT, line of therapy; MD, mean differences; mo, months; PBC, platinum-based chemotherapy; SD, standard deviation.

^a^MDs were calculated as the difference in mean costs. CIs were calculated based on the sample sizes for each LOT and SDs for each cost outcome using the following formula:

$95\%CI=\mathrm{MD}\pm 1.96\sqrt{\frac{SD_{A}^{2}}{n_{A}}+\frac{SD_{B}^{2}}{n_{B}}}$

^b^Duration of the LOT was defined as the time from the date of initiation of the LOT until the day preceding the initiation of the following LOT. Patients who did not initiate a next LOT were censored at the end of their observation period.

^c^Other costs included all services other than outpatient, inpatient, and emergency room services, including durable medical equipment as well as dental and vision care.

**Table 4.** All-cause Costs During Each LOT among Patients Treated With Osimertinib Monotherapy or PBC Without Immunotherapy in 1L

**Monthly All-Cause Total Healthcare Costs, 2022 USD, Mean ± SD [Median]**	**Costs During 1L Therapy**			**Costs During 2L Therapy**			**Costs During 3L Therapy**		
**Treated With Osimertinib Monotherapy in 1L therapy [A] (N = 279)**	**Treated With PBC Without Immunotherapy in 1L Therapy [B] (N = 58)**	**A vs B,^a^ MDs (95% CI)**	**Treated With Osimertinib Monotherapy in 1L Therapy [A] (N = 67)**	**Treated With PBC Without Immunotherapy in 1L Therapy [B] (N = 58)**	**A vs B,^a^ MDs (95% CI)**	**Treated With Osimertinib Monotherapy in 1L Therapy [A] (N = 33)**	**Treated With PBC Without Immunotherapy in 1L Therapy [B] (N = 33)**	**A vs B,^a^ MDs (95% CI)**	
Duration of LOT^b^ (mo), mean ± SD [median]	11.9 ± 10.7 [8.6]	5.9 ± 9.5 [3.4]		7.4 ± 8.9 [4.4]	10.0 ± 9.7 [6.4]		7.0 ± 4.8 [4.9]	9.4 ± 8.7 [7.1]	
Total healthcare costs	27 610 ± 26 718 [21 794]	23 820 ± 17 718 [21 657]	3790 (−1744; 9324)	35 501 ± 28 330 [30 223]	24 788 ± 23 603 [18 912]	10 713 (1607; 19 819)	36 618 ± 32 752 [28 503]	23 348 ± 17 055 [18 827]	13 270 (671; 25 869)
Medical costs	11 443 ± 26 673 [4792]	23 393 ± 17 391 [21 484]	−11 950 (−17 412; −6488)	28 477 ± 28 499 [20 815]	15 300 ± 24 743 [4863]	13 177 (3843; 22 511)	30 465 ± 29 712 [21 813]	13 787 ± 18 013 [7871]	16 678 (4823; 28 533)
Outpatient costs	5465 ± 8953 [3121]	20 950 ± 16 868 [16 786]	−15 485 (−19 952; −11 020)	22 270 ± 22 918 [19 082]	10 670 ± 16 470 [3064]	11 600 (4665; 18 533)	20 872 ± 23 241 [14 584]	11 625 ± 17 800 [4071]	9247 (−740; 19 236)
Antineoplastic drug + administration costs	182 ± 815 [0]	9763 ± 14 454 [4267]	−9581 (−13 302; −5860)	14 979 ± 23 666 [7932]	7095 ± 16 948 [0]	7884 (733; 15 035)	12 476 ± 20 689 [1233]	6150 ± 11 896 [0]	6326 (−1817; 14 469)
Other outpatient costs	5283 ± 8740 [2983]	11 187 ± 12 229 [8106]	−5904 (−9214; −2594)	7291 ± 8202 [3954]	3575 ± 7661 [1610]	3716 (933; 6499)	8396 ± 12 300 [3913]	5475 ± 15 573 [1265]	2921 (−3850; 9692)
Inpatient costs	5635 ± 24 565 [0]	2043 ± 4845 [0]	3592 (451; 6733)	5662 ± 12 071 [0]	4333 ± 18 647 [0]	1329 (−4273; 6931)	8934 ± 21 717 [0]	1963 ± 4931 [0]	6971 (−627; 14 569)
Emergency room + other costs^c^	344 ± 1041 [0]	399 ± 1646 [0]	−55 (−496; 386)	546 ± 1678 [0]	296 ± 1136 [0]	250 (−247; 747)	659 ± 2653 [0]	200 ± 465 [0]	459 (−460; 1378)
Pharmacy costs	16 166 ± 5451 [16 459]	427 ± 1542 [69]	15 739 (14 986; 16 492)	7024 ± 10 020 [708]	9488 ± 10 674 [8903]	−2464 (−6111; 1183)	6153 ± 6832 [3431]	9561 ± 7022 [10 659]	−3408 (−6751; −65)

Abbreviations: 1L, first-line; 2L, second-line; 3L, third-line; CI, confidence interval; HRU, healthcare resource utilization; LOT, line of therapy; MD, mean differences; mo, months; PBC, platinum-based chemotherapy; SD, standard deviation; USD, US dollars.

^a^MDs were calculated as the difference in mean costs. CIs were calculated based on the sample sizes for each LOT and SDs for each cost outcome using the following formula:

$95\%CI=\mathrm{MD}\pm 1.96\sqrt{\frac{SD_{A}^{2}}{n_{A}}+\frac{SD_{B}^{2}}{n_{B}}}$

^b^Duration of the LOT was defined as the time from the date of initiation of the LOT until the day preceding the initiation of the following LOT. Patients who did not initiate a next LOT were censored at the end of their observation period.

^c^Other costs included all services other than outpatient, inpatient, and emergency room services, including durable medical equipment as well as dental and vision care.

Among the subgroup of patients treated with osimertinib monotherapy in 1L therapy, inpatient and outpatient costs accounted for a similar proportion of total costs in 1L therapy (20.4% [$5635 PPPM] and 19.8% [$5465], respectively). Outpatient costs subsequently increased to 62.8% ($22 270) in 2L therapy with the higher total cost in 3L therapy being driven by an increase in both inpatient and outpatient costs (24.4% [$8934] and 57.0% [$20 872] of total costs, respectively). Pharmacy costs in this subgroup were the highest in 1L therapy ($16 166 PPPM), representing 58.6% of the total costs, and subsequently decreased to 19.8% ($7024 PPPM) and 16.8% ($6153 PPPM) in 2L and 3L therapies, respectively.

Among the subgroup of patients treated with PBC without immunotherapy in 1L therapy, most of the total costs in 1L therapy were due to outpatient costs (88.0%; $20 950 PPPM), while inpatient costs accounted for only 8.6% ($2043). Outpatient costs subsequently decreased in 2L and 3L therapies, accounting for 43.0% ($10 670 PPPM) and 49.8% ($11 625 PPPM) of the total costs, respectively. Pharmacy costs for this subgroup were relatively marginal in 1L therapy ($427 PPPM) and increased to $9488 and $9561 in 2L and 3L therapies, respectively, representing 38.3% and 40.9% of the total costs in each respective LOT.

## DISCUSSION

In this real-world study of patients with EGFRm (Ex19del/L858R) advanced NSCLC initiating 1L therapy, progression to later LOTs was associated with numerically higher total healthcare costs and shorter LOT duration. Although data on the clinical benefits of osimertinib have established it as a frequently used 1L treatment for patients with EGFRm (Ex19del/L858R) advanced NSCLC, this study shows that patients treated with osimertinib monotherapy in 1L therapy had on average 1.0 inpatient admission per patient per year (PPPY) during 1L therapy, 1.6 inpatient admissions PPPY during 2L therapy, and 1.2 inpatient admissions PPPY during 3L therapy. Despite being an oral medication, patients treated with osimertinib monotherapy in 1L therapy had an average of 4 days PPPM with outpatient services. After 1L therapy, approximately 1 in 10 patients received immunotherapy in later LOTs, which is not guideline-recommended, highlighting the unmet medical needs for patients with EGFRm (Ex19del/L858R) advanced NSCLC who progress on treatment.

The HRU and all-cause healthcare costs incurred by patients with EGFRm (Ex19del/L858R) advanced NSCLC in 1L therapy reported in the current study are in line with the findings of Vanderpoel et al.^12^ Consistent with the findings of the current study, Vanderpoel et al^12^ observed that patients treated with osimertinib in 1L therapy had numerically more inpatient days and incurred numerically higher inpatient costs during 1L therapy, while chemotherapy-treated patients incurred numerically higher outpatient costs in 1L therapy, likely owing to the additional outpatient visits required for chemotherapy infusions (in the current study, patients in the PBC without immunotherapy subgroup had a mean of 8 days with outpatient services PPPM relative to 5 days in the overall cohort and 4 days in the subgroup of patients treated with osimertinib monotherapy in 1L therapy). While Vanderpoel et al^12^ only reported outcomes for 1L therapy, the current study builds on this by showing that patients who were treated with osimertinib monotherapy in 1L therapy continued to have high total healthcare costs in subsequent LOTs, driven by both high inpatient and outpatient costs. Further research is needed to determine the underlying drivers for the increased HRU and costs among patients with EGFRm (Ex19del/L858R) advanced NSCLC treated with osimertinib monotherapy in 1L therapy.

The therapeutic value of anticancer therapies should encompass their ability to effectively treat a condition while ideally limiting HRU, reducing the impact of the condition on the patient and alleviating the economic burden on payers and healthcare systems. Previous studies have demonstrated the superior efficacy and tolerability of osimertinib compared with other EGFR-TKIs as well as PBC,^7,10,15^ which is reflected in the numerically longer average duration of 1L osimertinib monotherapy in the current study (11.9 months) relative to the duration of 1L PBC without immunotherapy (5.9 months). However, the numerically longer duration of hospitalizations and average of 4 outpatient visits per month observed in this real-world study among patients treated with osimertinib monotherapy in 1L therapy may be reflective of unmet needs for these patients and could have implications for disease management, clinical outcomes, and patient quality of life in addition to the increased healthcare cost associated with inpatient visits. Moreover, outpatient costs unrelated to antineoplastic administration increased by LOT among patients who received osimertinib monotherapy in 1L therapy.

In addition to evaluating HRU and costs among patients with EGFRm (Ex19del/L858R) advanced NSCLC, this study provided a description of their treatment sequences from 1L to 3L therapy. Results show that despite significant advances in targeted treatments, a notable proportion of patients continue to receive PBC with or without immunotherapy as 1L therapy and progress quickly to 2L therapy. Moreover, after 1L therapy, 1 in 10 patients received immunotherapies, which are not guideline-recommended for patients with EGFRm (Ex19del/L858R) advanced NSCLC in later LOTs as they are less effective.^16^ Owing in part to the heterogeneity of NSCLC, resistance to EGFR-TKIs, which can be polyclonal, is inevitable.^17,18^ As such, the optimal use and sequence of therapies for patients with EGFRm (Ex19del/L858R) advanced NSCLC continues to be a topic of investigation since selecting subsequent therapies in clinical practice at the time of progression can be complex.^7,8,19^ Together, these results highlight the unmet needs among patients with EGFRm (Ex19del/L858R) advanced NSCLC for effective 1L therapies that are better capable of balancing clinical benefits and safety risks with overall HRU.

The current study focused on evaluating the real-world outcomes of patients with EGFRm (Ex19del/L858R) advanced NSCLC, as the EGFR-TKIs investigated were approved by the US FDA specifically for this population. However, the molecular heterogeneity of *EGFR* mutations, which can have important implications for treatment response, as some may be associated with de novo resistance,^20,21^ warrants additional consideration. Specifically, EGFR-TKIs have been shown to have limited efficacy for patients with exon 20 mutations, which is the third most common mutation in this patient population.^20,21^ Therefore, novel medications treating a variety of mutation subtypes are needed. Additionally, among patients with EGFRm (Ex19del/L858R), 1L treatment with combination regimens such as amivantamab plus lazertinib and osimertinib plus chemotherapy have recently shown longer progression-free survival relative to osimertinib monotherapy in clinical trials,^22,23^ and warrant further investigation in real-world settings.

### Limitations

Some limitations of this study should be considered when interpreting the results. The study was descriptive in nature and as a result, there were no adjustments for potential confounders or statistical comparisons conducted between subgroups or across LOTs. The reason for hospitalizations and outpatient visits, including whether they were related to the treatments received, was also not available in the current study. Additionally, the costs evaluated in this study only included the direct healthcare costs captured in claims data and did not consider indirect costs. Furthermore, information related to mortality was not available in the data source and, as such, the impact of death on study outcomes, including costs associated with end-of-life care, could not be assessed. Inherent to claims-based studies, data may have contained possible inaccuracies due to coding errors and missing data. Given that there are no diagnosis codes specific to NSCLC, an algorithm was used to select these patients based on medication use and/or metastatic diagnosis; however, based on this algorithm, more than 90% of patients had a metastatic diagnosis code within 30 days of the first LC diagnosis, which may have led to the inclusion of a later-stage population. In the absence of staging and mutation status in administrative claims data, treatments indicated for EGFRm (Ex19del/L858R) advanced NSCLC were used as a proxy for patients with EGFRm (Ex19del/L858R) advanced NSCLC. However, some patients included in the final study sample may not have a EGFRm (Ex19del/L858R) advanced NSCLC despite initiating a treatment indicated for patients with EGFRm (Ex19del/L858R) advanced NSCLC. Similarly, the study excluded patients who may have had *EGFR* mutations but were not treated with an *EGFR*-TKI. The final study sample may have also included patients with *EGFR* exon 20 insertions who were inappropriately treated with EGFR-TKI (ie, to which they are known to be resistant)^20,21^ and who may have a higher HRU and cost burden than patients with Ex19del/L858R mutations.^24^ Finally, although the study may have included some patients who initiated 1L therapy as adjuvant therapy following LC-related surgery or radiation, the objective of the study was to present the unmet needs and economic burden among patients treated with *EGFR*-TKIs in real-world clinical practice, which includes patients who receive LC-related surgery or radiation prior to initiating 1L therapy with a treatment regimen for advanced NSCLC. To minimize the possibility of capturing adjuvant therapy in the current study, patients without a diagnosis for metastatic disease within 30 days following their first LC diagnosis who had at least 1 claim for LC-related surgery in the period of continuous pharmacy and medical insurance eligibility prior to 1L treatment initiation were excluded.

## CONCLUSIONS

Patients with EGFRm (Ex19del/L858R) advanced NSCLC experience high HRU and inpatient costs, including those treated with osimertinib monotherapy in 1L who continue to require multiple outpatient visits per month despite its oral mode of administration and longer duration of therapy relative to PBC. Furthermore, subsequent disease progression is associated with numerically higher total costs of care and the use of less effective therapies as approximately 1 in 10 patients received treatments that are not guideline-recommended (ie, immunotherapy) in second or later LOTs. Recently approved therapies such as 1L combination regimens may delay costly disease progression in patients with EGFRm (Ex19del/L858R) advanced NSCLC and warrant further research to evaluate their associated HRU and costs.

### Disclosures

D. Waterhouse reports personal consulting fees from Amgen, Astellas Pharma, AZTherapies, Bristol-Myers Squibb, Eisai, Fresenius Kbi, Gilead Sciences, Johnson & Johnson, Lilly, Merck, Mirati Therapeutics, Novartis, Pfizer, Regeneron/Sanofi, Sanofi, and Takeda, as well as the speakers’ bureau for Amgen, AZTherapies, Bristol-Myers Squibb, EMD Serono, Fresenius Kbi, Johnson & Johnson, and Merck. I.L., P.V., and D. Waters are employees and stockholders of Johnson & Johnson. L.M., B.E., A.H., J.K, and P.L. are employees of Analysis Group, Inc., a consulting company that was provided research funding from Johnson & Johnson, which funded the development and conduct of this study and manuscript. M.-H.L. was an employee of Analysis Group, Inc. at the time of the study.

### Previous Presentations

Part of the material in this manuscript was presented in a poster at the Academy of Managed Care Pharmacy (AMCP) Annual Meeting held April 15-18, 2024, in New Orleans, Louisiana.

## Supplementary Material

Online Supplementary Material

## Data Availability

The data that support the findings of this study are available from IBM® MarketScan®, but restrictions apply to the availability of these data, which were used pursuant to a data use agreement. The data are available through requests made directly to IBM®, subject to applicable requirements for data access.
