# Supplementary material for: Healthcare Resource Use, Healthcare Costs, and Unmet Needs Among Patients Treated for EGFR-Mutated Advanced or Metastatic Non-small Cell Lung Cancer
Source: J Health Econ Outcomes Res. 2025 Aug 29;12(2):98–107. doi: 10.36469/001c.142771 (PMC12399230; doi:10.36469/001c.142771)
Supplement: Online Supplementary Material [file jheor_2025_12_2_142771_300045.pdf]

Journal of Health Economics and Outcomes Research

Online Supplementary Material

Healthcare Resource Use, Healthcare Costs, and Unmet Needs Among Patients Treated for EGFR-Mutated Advanced or Metastatic Non-small Cell Lung Cancer. *JHEOR*. 2025;12(2):98-107. doi:10.36469/jheor.2025.142771

List of Guideline-Recommended Treatments for Advanced Non-Small Cell Lung Cancer

Figure S1: Sample Selection

This supplementary material has been provided by the authors to give readers additional information about their work.

This is an open-access article distributed under the terms of the Creative Commons Attribution 4.0 International License (CCBY-4.0). View this license's legal deed at http://creativecommons.org/licenses/by/4.0 and legal code at http://creativecommons.org/licenses/by/4.0/legalcode for more information.

List of Guideline-Recommended Treatments for Advanced Non-Small Cell Lung Cancer

Adagrasib
Ado-trastuzumab emtansine
Afatinib
Alectinib
Amivantamab-vmjw
Atezolizumab
Bevacizumab
Brigatinib
Cabozantinib
Capmatinib
Carboplatin
Cemiplimab
Ceritinib
Cisplatin
Crizotinib
Dacomitinib
Dabrafenib
Docetaxel
Durvalumab
Erlotinib
Entrectinib
Fam-trastuzumab deruxtecan-nxki
Gefitinib
Gemcitabine
Ipilimumab
Larotrectinib
Lorlatinib
Mobocertinib
Nivolumab
Osimertinib
Paclitaxel
Pembrolizumab
Pemetrexed
Pralsetinib
Ramucirumab
Selpercatinib
Sotorasib
Tepotinib
Trametinib
Tremelimumab-actl
Vemurafenib
Vinorelbine

Figure S1. Sample Selection

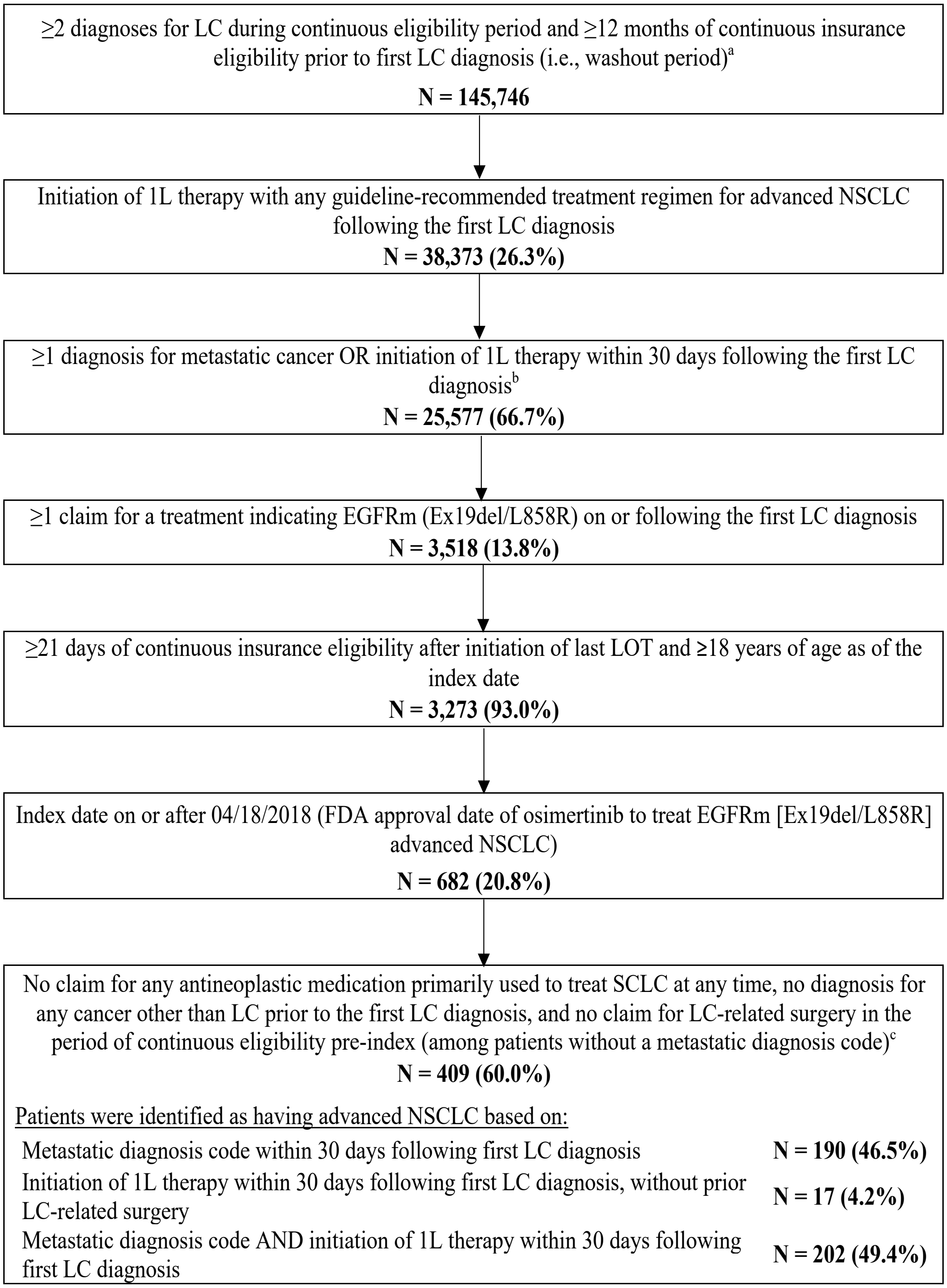

Abbreviations: 1L, first line; EGFRm, epidermal growth factor receptor mutations; Ex19del, exon 19 deletions; FDA, Food and Drug Administration; ICD-9/10-CM, *International Classification of Diseases, Ninth/Tenth Revision, Clinical Modification*; L858R, exon 21 L858R substitutions; LC:,lung cancer; (N)SCLC, (non-) small cell lung cancer.

Notes:

[a]Diagnoses for LC were identified based on the following ICD-9-CM codes: 162.2x-162.9x and ICD-10-CM codes: C34.x.

[b]Diagnoses for metastatic cancer were identified based on ICD-9-CM codes: 196.x, 197.x, 198.x, 209.7 and ICD-10-CM codes: C77.x, C78.x, C79.x, C7B.x.

[c]Antineoplastic treatments used to treat SCLC included: etoposide, irinotecan, and topotecan; LC-related surgeries included lung lobectomy, lung resection, segmentectomy, and pneumonectomy.
